# Facilitators and Barriers for Participation in Physical Activity Among Norwegian Physically Active First-Year Students: A Qualitative Study

**DOI:** 10.3390/ijerph23050673

**Published:** 2026-05-19

**Authors:** Friedolin Steinhardt, Stine Pedersen Bøtun, Line Dverseth Tjærandsen

**Affiliations:** 1 Department of Health and Nursing Sciences, Faculty of Social and Health Sciences, University of Innland Norway, 2406 Elverum, Norway; 2 Department of Physical Education, Sports, and Outdoor Life, Faculty of Education and Arts, Nord University, 8026 Bodø, Norway; stine.pedersen@nord.no (S.P.B.); line.d.tjarandsen@nord.no (L.D.T.)

**Keywords:** physical activity, COM-B, university students, facilitators, barriers, Norway

## Abstract

**Highlights:**

**Public health relevance—How does this work relate to a public health issue?**

- Physical inactivity is prevalent among university students despite its well-documented risks.
- The transition to university life represents a critical period for establishing long-term health behaviours, including physical activity habits.

**Public health significance—Why is this work of significance to public health?**

- This study provides in-depth qualitative insight into how capability, opportunity, and motivation interact to influence physical activity among first-year students.
- Experiences from already-active students are used to help identify factors that sustain physical activity, an underexplored area in public health research.

**Public health implications—What are the key implications or messages for practitioners, policymakers, and/or researchers in public health?**

- Universities should implement integrated strategies addressing structural, social, and psychological factors to promote sustainable activity habits.
- Enhancing social support, access to facilities, and students’ self-regulation and competence may strengthen long-term engagement in physical activity.

**Abstract:**

Regular physical activity is essential for physical and mental health, yet participation among Norwegian university students remains below nationally recommended levels. This study explored facilitators and barriers for physical activity among first-year students, using the COM-B model as a conceptual framework. Fifteen physically active first-year students from two higher education campuses in Bodø were interviewed in spring 2025, and the data were analysed using inductive thematic analysis. Analysis showed that students’ activity behaviours were shaped by a dynamic interaction between physical and psychological capabilities, particularly in relation to technical competence, previous injuries, and self-regulation strategies. Opportunity-related factors—such as time constraints, financial limitations, commuting distance, and access to facilities—substantially influenced students’ ability to maintain regular activity, while social support from friends, family, and peers functioned as an important facilitator. Motivation emerged through a mixture of automatic processes—including stress reduction, enjoyment, and habits—and reflective processes such as goal-setting and health-oriented decision-making. For students in physically demanding study programmes, professional identity and body-related expectations also contributed to their engagement. Overall, this study highlights the need for institutional strategies that simultaneously address structural, social, and psychological factors to support sustainable physical activity habits during the transition to university life.

## 1. Introduction

There is a large consensus among scientists on the benefits of regular physical activity (PA) to both physical and mental health, as well as overall well-being [1,2,3]. Concurrently, the negative effects of physical inactivity are also well-established, including a higher risk of noncommunicable diseases (NCDs) [4]. According to the World Health Organization, physical inactivity is projected to add a worldwide economic burden of 27 billion USD to healthcare systems each year from 2020 to 2030 [5]. It is responsible for between 26% and 41% of preventable cases of NCDs in lower-middle to high-income countries [3]. In a pooled analysis of 358 surveys with 1.9 million respondents worldwide, Guthold and colleagues [6] found that approximately 27.5% of respondents fall under the category of insufficient PA. In Norway, health authorities recommend 150 min of moderate or 75 min of vigorous activity—or a combination of both—each week for adults [7].

Norway represents a distinctive context for physical activity. A lot of activity happens in nature, due to the *friluftsliv*-concept, a culturally embedded orientation towards outdoor recreation, supported by widespread access to natural environments and egalitarian welfare structures that reduce economic barriers to participation [8,9]. At the same time, participation patterns reflect seasonal variability, due to extended periods of darkness during the winter and longer days during the summer [10].

Three in four Norwegian adults and older adults fulfil these recommendations [11]. However, according to a national study by Grasdalsmoen and colleagues [12], only 23.3% of male and 17.9% of female college and university students meet these minimum criteria for PA. At the same time, 39.7% of female and 25.7% of male Norwegian students fulfil the criteria of at least one mental health disorder [13], and the proportion of overweight and obese students has also risen from 23% (29% for male and 19% for female) in 2010 to 37% (41% for male and 35% for female) in 2022 [14].

In a meta-analysis, Winpenny and colleagues [15] showed that the transition from high school to college or university leads to an average decrease of 7 min in moderate to vigorous physical activity (MVPA) per day, with a greater decrease in males than females. This is consistent with international research that characterises this transition as a pivotal period in life, in which new habits and health behaviours form and will likely affect health outcomes throughout life [16,17,18].

In order to prevent inactivity and the subsequent increased risk of NCDs and mental health disorders, interventions designed to increase PA among the student population should be a focus of health promotion work at colleges and universities [16,18]. However, efficient intervention design requires a deeper understanding of factors influencing PA-related behaviour of Norwegian students [17,19].

To form a comprehensive understanding of health-related behaviour, Michie and colleagues [19] designed ‘the Behavioural Change Wheel’, also known as the COM-B model. The main hypothesis of the model states that, for any given behaviour to occur, an individual needs capability (both physical and psychological), opportunity, and motivation to engage in the behaviour [19]. Willmot and colleagues [18] conducted an empirical examination of the model with young adults in Australia between the ages of 18 and 35, focusing on both PA (n = 582) and nutrition (n = 455). Motivation was shown to be positively associated with PA, with both identity, positive affect, self-efficacy, and intentions being significant indicators for students’ motivation. A positive association was also found between capability and PA, with motivation having a mediating effect; habits, action control, and action planning were significant indicators for capability. Opportunity was also positively associated with PA—again mediated by motivation—with social support and subjective norms being significant indicators. Furthermore, environments favourable to PA further increased the motivation of young adults to be physically active. Recently, Brown and colleagues [16] conducted a systematic review of the key influences on PA among university students, summarising 39 studies. The five most influential domains on students’ activity were ‘environmental context and resources’, ‘social influences’, ‘goals’, ‘intentions’, and ‘reinforcement’. The first two of these domains represent areas of opportunity in the COM-B framework, and the latter three represent motivation. According to the same analysis, the most frequent barrier for engaging in PA was a lack of time, mostly due to university-related tasks, long commutes, family responsibilities, or employment commitments. Other environmental barriers reported in the systematic review were financial costs associated with memberships, equipment, or access to exercise facilities; a lack of personalised PA to cater to individual needs; a lack of policies to promote PA at the university; and health-related behaviours associated with student life (e.g., poor diet, increased alcohol intake, or increased sedentary behaviour). Sociocultural norms, in which PA was deemed an important part of the culture, were reported to facilitate engagement in PA. In terms of motivation, exercising with others increased accountability and enjoyment among students, as did encouragement and positive reinforcement from others. Conversely, a lack of friends to exercise with or a lack of social support were commonly cited barriers. Prioritising PA over other leisure activities facilitated participation, especially for team sports. Furthermore, students involved in competitive sports and those who set goals related to their performance seemed more motivated and engaged in PA; meanwhile, external goals, such as improving appearance, were less effective in facilitating PA.

Although different studies have looked at general student populations [20,21,22,23] or specifically focused on inactive students [24], little research has focused on active students. Although knowledge on inactive or insufficiently active students is of importance from a public health perspective, it leaves a critical gap in our understanding of how PA is maintained among those who successfully remain active during the transition to university. Brown et al. [16] explicitly highlight that much of the literature identifies barriers such as lack of time, competing academic demands, and limited access to facilities—but provides far less insight into how some students manage to sustain regular PA, despite these constraints.

We propose that knowledge of factors that facilitate the engagement of active students in PA, as well as barriers perceived by this group, could inform policymakers and actors in higher education about protective mechanisms to maintaining activity in a high-risk transition and implementing interventions to enhance PA. First-year students represent a distinct phase in the student life course, characterised by cultural adjustment, the establishment of new routines, and increased demands for self-regulation. In contrast to students in later years, first-year students are simultaneously adapting to academic expectations, social environments, and independent health-related decision-making. The aim of this study was therefore to investigate the facilitators and barriers perceived by active Norwegian first-year students. Since we aimed to investigate the individual perspectives of these students, a qualitative study-design was adopted, utilising individual interviews.

## 2. Materials and Methods

### 2.1. Design and Context

This sub-study is part of the larger research project *“På egne bein” (‘On your own feet’)*, which aims to examine how the transition to student life affects the lifestyle and overall health of students throughout their first year of study. The project targets first-year students at Norwegian colleges and universities in the Agder, Inland, and Nordland County.

### 2.2. Sample and Inclusion Criteria

**Figure 1:**
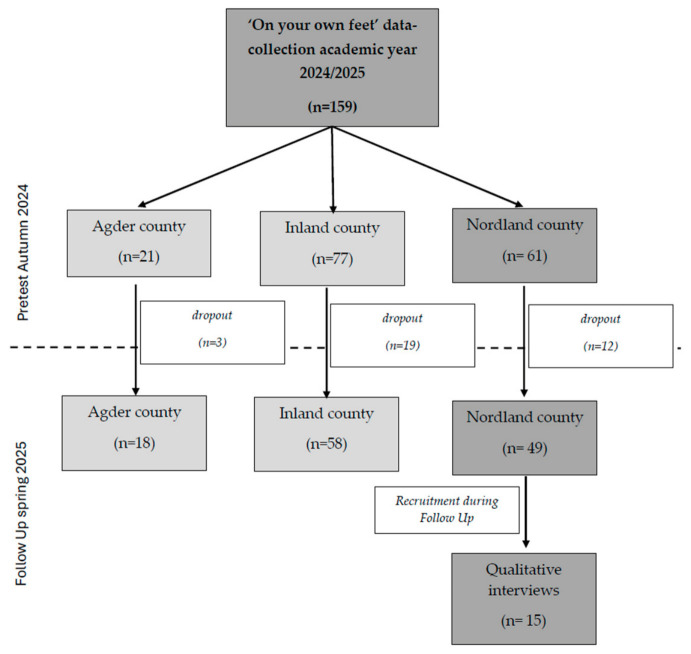
Flowchart for the recruitment of the sample.

Participants in the main project had to be first-year students at colleges or universities in Norway, be under 22 years of age, and be enrolled in higher education for the first time. For this sub-study, 61 first-year students attending the Bodø campuses of Nord University and the Norwegian Police University College in autumn 2024 were defined as the sampling base. Of these, 15 participants were recruited for qualitative interviews in spring 2025. A strategic sampling approach was employed to ensure inclusion of participants of both sexes and from different study programmes. Although the original intention was to include both physically active and inactive first-year students, only students who were already physically active ultimately participated in the study. Recruitment was discontinued when data saturation was reached and no new relevant information emerged from additional interviews. Figure 1 shows the recruitment of the sample within the data collection of the ‘On your own feet’ project for the academic year 2024/2025.

### 2.3. Data Collection

Participants were contacted during data collection for the main project and asked if they wished to participate in a qualitative interview. Interviews were conducted in April/May 2025, at the end of the teaching period of the academic year. The interviews were conducted in a group room at Nord University and lasted between 25 and 45 min. All three authors (two of them with experience of conducting interviews on a Ph.D. level) conducted the interviews. To ensure consistency in the interview process the first two interviews were conducted by to researchers, with one interviewing and one observing, followed up by discussing interview techniques and themes to follow up on.

A semi-structured interview guide was used [25], beginning with the researcher introducing themselves and explaining their academic position and relation to the project, in case they have not met the participant earlier through recruitment or data-collection of the main study. This was followed up by introductory questions about the participants’ backgrounds. The main topics in the guide were related to barriers to and facilitators of PA. Table 1 gives an overview over the main topics and questions in the interview guide.

Although all participants were asked questions within the same thematic areas, the order of questions was flexible and adapted to the natural flow of the conversation. Further follow-up questions were tailored to the information provided in each interview to allow for in-depth exploration of participants’ experiences, rather than relying on fixed prompts for barriers, facilitators. This approach allowed participants to emphasise what they considered most relevant. All interviews were audio-recorded, using the ‘Nettskjema Dictaphone’ application [26]. Recordings were transcribed verbatim with the help of AI and corrected by the authors.

### 2.4. Analysis

The data were analysed using thematic interpretative analysis [27]. The analysis followed an inductive approach, allowing themes to emerge from the material. At each stage, three researchers conducted independent analyses and discussed the findings until consensus was reached.

The analysis began with an initial, independent reading of the interview transcripts by each researcher to gain familiarity with the material and identify recurring patterns in the material. During this phase, preliminary themes were noted, and potential categories for a thematic analysis were discussed among the researchers. This was followed by several iterative rounds of coding based on the agreed thematic categories. The individual analyses were then merged and systematically compared through joint analytic meetings to discuss similarities, discrepancies, and interpretations across researchers. In a final analytical step, the established themes were mapped onto the COM-B framework and interpreted in relation to the definitions of capability, opportunity, and motivation as outlined by Michie and colleagues [19]. In this process, several of the initial themes identified through thematic coding were combined. For example, the themes *‘Prioritisation due to a tight time schedule’*, *‘Study-related commitments’*, and *‘Everyday commitments’* were merged into a single category labelled *‘Prioritisation due to time constraints and competing obligations’.* Table 2 exemplifies the analysis process.

### 2.5. Ethical Considerations

The study was approved by the Regional Committee for Medical and Health Research Ethics (REK; reference number: 255367) and the Norwegian Agency for Shared Services in Education and Research (SIKT; reference number: 607446). Nord University was the data controller for personal information. All participants received written and oral information about the study and signed informed consent prior to the interviews. It was emphasised that participation was voluntary and that participants could withdraw at any time without consequences. Participants’ anonymity was maintained throughout the process, and publication will be carried out in a way that prevents the identification of individuals. All participants were assigned randomized fictitious names to ensure anonymity in reporting.

## 3. Results

The material of this study is based on interviews with 15 first-year students (7 male; 8 female), aged 20 to 23 (22.9 ± 0.8), from six different study programmes, including police, personal training, sports science, aquaculture, social work, and animal care. All informants were enrolled in higher education for the first time.

This section presents findings from the qualitative interviews, organised according to the COM-B framework of the Behaviour Change Wheel: Capability, Opportunity, and Motivation [19]. Within each domain, we report subthemes and illustrate them with verbatim quotations. Table 3 gives an overview over the different subthemes within the different domains of the COM-B framework:

### 3.1. Capability

Capability encompasses both physical and psychological skills that influence the ability to engage in PA [19].

### 3.2. Physical Capability

Physical capability relates to an individual’s physical capacity to engage in PA [19]. Several students described how injuries or illness disrupted established routines and reduced their physical capacity. One participant illustrated his experience as a disruptive event breaking his established activity routines, leading to a loss of momentum and difficulties resuming physical activity.

*“When I got sick, I had to quit for a while. Unfortunately, I never started again. [...] If I have two weeks where I do nothing, I lose that… yes, the flow, that’s what I call it. So that’s quickly the problem for me at least.”*

Leif

Overload and chronic conditions required adaptation of exercises and intensity for several students. One participant explained necessary adaptations due to a chronic musculoskeletal condition:

*“With strength training, I really have to adapt the type of exercises I do. […] Because I don’t want to show myself that I’m handicapped. I would much rather show that I can do it.”*

Emil

### 3.3. Psychological Capability

Psychological capability describes an individual’s mental capacity to engage in PA; it includes knowledge, cognitive skills, and decision–making processes [19]. Students had varying perceptions of what constitutes a high level of activity, often shaped by family habits and previous experiences. One participant student distinguished clearly between structured, sport-based physical activity and lifestyle-oriented outdoor activities, initially perceiving the family as “not active” despite frequent engagement in hiking and skiing. This suggests that organized sports were central to the student’s understanding of what counts as being active, shaping a narrow but intense conceptualization of physical activity.

*“But mom and dad aren’t that active. […] Sport-wise, we didn’t do much together, really. Me and my mom used to go swimming once a week, maybe. Besides that... Yes, we went on a lot of cabin trips and stuff like that, both Easter and winter. And we went hiking and went downhill skiing and stuff. Downhill skiing was actually a big part of my life, like that... when I was young—or younger. When I was a child. Then there was a lot of slalom, actually. It was like every winter holiday and every Easter holiday, then it was downhill skiing [...]”*

Sondre

This reflects socially shaped psychological capability, where family norms emphasized organized sport over lifestyle activities, thereby influencing what the student perceived as legitimate or visible physical activity.

When reflecting on their upbringing, several participants had similar reflections, in which they began by stating that their family or parents were not particularly physically active, followed by an extensive list of PAs they did together.

Multiple participants mentioned that a lack of instruction was linked to poor technique and injuries, while structured education strengthened technical competence.

One participant explained how she acquired injuries early in her training due to a lack of competence.

*“I injured my knees, because I started out wrong when I began my training journey.”*

Tiril

On the other hand, another student explained how joining the sports programme at her high school helped her to gain more competence in strength training.

*“Then I started at a sports programme, and we had a weight room there. […] That’s when I learned more technique for squats and deadlifts.”*

Alma

Several emphasised self-management and prioritisation as part of capability and taking responsibility for their own PA. One student explained his experiences of self-organised physical activity as a process of full autonomy accompanied by personal responsibility and pressure due to the absence of external structure or supervision, going so far as to calling himself his own coach:

*“I’m like my own coach; I plan the sessions myself, execute them myself, no one is watching me, so all the pressure is on my shoulders.”*

Sondre

The transition to student life required planning and structuring of time, as one participant explained:

*“It was difficult, in a way, figuring out how to establish myself. […] Then I’ve been able to train a lot more than I might have done at the start, because then there were a lot of impressions you had to take in, and the information you had to take with you.”*

Bjørn

Comments like these show how organising physical activity might initially be limited psychological capability, due to the need to navigate new contexts student life and large amount of information but gradually develops greater capacity for training as familiarity and structure are established.

### 3.4. Interaction Between Physical and Psychological Capability

Several students explained how previous injury experiences provided them with strategies to physically adapt their training due to acquired knowledge. At the same time, this led them to better cope with the situation on a mental level, as exemplified by the comment from one young woman:

*“The injury wasn’t really new either. The one that came this fall… It was an old injury, and I know what to do to make it better.”*

Ingrid

Others explained how they used equipment or modified exercises to compensate for limitations, which in turn strengthened their perception of their control and competence:

*“First of all, it felt good to know that things stayed in place without me having to concentrate on it. It was a dream. It was pure bliss.”*

Emil

### 3.5. Opportunity

Opportunity includes external factors that influence students’ practical and social conditions for PA [19].

### 3.6. Physical Opportunity

Physical opportunity relates to external and environmental factors that might enable engagement in PA [19]. Environmental factors affected students’ practical ability to be active. Access to facilities and campus provisions was highlighted as a resource by several students, especially those studying in programmes that included free access to training facilities, such as sports science and police. One participant explained:

*“At the police academy, we have our own gym in the basement, which is free for us. So I think that’s a really good offer.”*

Aksel

Another student commented on the wide variety of sporting facilities available for students:

*“It’s very good, we have a running track, a gym at school, a volleyball court, and a football pitch. So it’s been awesome, very pleasantly surprising.”*

Ingrid

From a COM-B perspective, the students’ positive experiences reflect strong physical opportunity, as accessible, on-campus facilities and free access reduce structural barriers to physical activity.

Similarly, the availability and variety of different organised activities, courses, and clubs at the university were perceived as positive and facilitating PA. One participant further highlighted the welcoming and inclusive nature of these activities, including free trials, further facilitating physical opportunity:

*“I think it’s very good. You have everything from the start of your studies, when you have that trial week. You can participate in whatever you want, and see what you like. [...] Everyone finds something they want to be part of. I signed up for football alongside others. There is room for 20 people. We usually have like 15–16 showing up for training. They are very good at planning and facilitating that those trainings are actually carried out during the week.”*

Bjørn

At the same time, distance to training venues was a challenge, especially for students living on the other side of the town, as one participant explained:

*“I live on the opposite side of the city compared to the school. So it’s a bit far to travel when I don’t have a car.”*

Ronja

Financial constraints limited participation in certain activities, as one student described her choice of memberships due to financial restrictions:

*“I’ve put climbing on hold because it’s a half-year subscription, which I chose because it was the cheapest. […] I will start again next spring.”*

Signe

At the same time, several students reported prioritising spending money for training or gym memberships—even with free access to facilities on campus—to get more variety in their training:

*“We at the police academy have our own gym down in the basement, which is also free for us. I think it’s a very good offer. [...] I also have a membership at another gym. In principle, I don’t need a membership there. But I have it for variety. I get a little tired of the facilities, equipment, and such.”*

Sondre

Despite having free access to a campus gym, the pursuit of variety indicates that diverse facilities are perceived as necessary to sustain effective training and bodily competence for this student and reflects a need to support physical capability through varied equipment and training environments, accepting additional costs.

Season and weather were mentioned as practical barriers for physical capability. One of the main factors mentioned to affect the student’s motivation, in this regard, was the extensive darkness:

*“Yes, the motivation drops quite a bit, when it’s so grey and sad.”*

Ingrid

Further, time pressure and competing demands were recurring challenges, as one participant explained, with regard to higher demands during the exam period:

*“During exam periods, there’s so much schoolwork, so you’re more tired, and then I didn’t have the energy to train much.”*

Signe

Finally, the combination of different demands and competing needs also affected the students and forced them to prioritise—sometimes leading them to deprioritise additional sports activities and training:

*“Yes, I mean, when I take into account that we have school, and then you want to make yourself some food, and then I want to fit in a workout session, and then maybe have a few small snacks in between, and there’s also some reading and schoolwork to do, you also want a bit of time to just relax, lower your shoulders, and rest on the couch. So I’ve simply decided that there isn’t really time for that in my everyday life, and I’m quite satisfied with the daily routine I’ve set up.”*

Einar

The student clearly experiences competing demands from his commitments in academics, daily live, and recovery as challenging physical capability, requiring careful regulation of energy, time, and bodily capacity.

### 3.7. Social Opportunity

Social opportunity relates to an individual’s social environment and how it influences participation in PA, including social norms, cues, or support from others [19]. Social relationships and support from family and friends were crucial for students’ activity habits. Family and upbringing were often highlighted as a resource:

*“An active childhood. With football and football. The whole family played football in the same club.”*

Ingrid

This also included parental support, which allowed students to try out different activities and helped form an active lifestyle:

*“If I wanted to do something, they [my parents] supported me in it. And joined me... They’ve really supported me.”*

Linnea

Friends and training environments contributed to a sense of community and enhanced motivation in addition to social opportunity. Several participants highlighted the importance of the social environment at the gym, and students supporting each other was particularly beneficial in the beginning:

*“I feel, especially in the start, it’s extremely important. Because then you get to exercise and be social at the same time. And then it goes a little faster with the training too. Thus, this is particularly important.”*

Ingrid

At the same time, other students prioritised independent training, using it as personal “me time”, as illustrated by one participant who lived in a large collective:

*“I thrive very much in my own company. When I’m training, it’s perhaps the only place where I feel like I have some ‘alone time’. Considering that I live in a collective, and in addition spend my days at school surrounded by many different students and friends, and so on.”*

Sondre

This specific case exemplifies how a highly saturated social environment—large, shared housing and constant peer interaction—shapes social opportunity by limiting access to solitude in everyday life. Physical activity becomes a compensatory context that enables temporary withdrawal from social demands, thereby supporting engagement through the creation of a valued social boundary rather than social interaction.

Another facilitator for training was the increased motivation experienced by students by having friends or training partners pushing each other in the gym:

*“I’ve trained with people I attend school with. So, for example, when we run intervals, I find it much more motivating to someone beside me. Because then I get a little extra push to complete the session. The same applies to strength training, really.”*

Alma

Another informant explained specifically how students would motivate each other during strength training:

*“It can seem more motivating, because you kind of have someone there. If they see that you’re lifting heavy, they come over, and then you push yourself to do one more repetition. So the whole training environment we have developed through the study program is really what makes it fun for me.”*

Freja

These examples illustrate how shared training contexts enhance social opportunity by providing peer presence, encouragement, and social comparison that stimulate greater effort and persistence. The sense of community within the study programme creates a supportive social environment that transforms physical activity into a socially rewarding and motivating experience.

Several students further mentioned how being in a general environment surrounded by active people enhanced their own motivation to be active:

*“When you are around people who train all the time, you get completely hooked on it yourself.”*

Bjørn

### 3.8. Interaction Between Physical and Social Opportunity

Several students noted that the physical demands of their study programme led them to prioritise their training:

*“You have to pass certain physical requirements. You have physical tests to get in [the study programme], and you have physical tests in the third year that you get grades on. So it’s definitely a factor to be in good physical shape.”*

Aksel

Others emphasised that their physical condition would affect their performance in their future work as police officers, as one participant explained:

*“It’s an advantage to be in good physical shape, because you must be strong to put someone on the ground, or run after someone, and things like that. But also, because if you have more physical capacity, then you have a greater capacity in general, to handle stressful situations [...] So I think it’s very important in a job context as well.”*

Alma

The students’ accounts highlight how institutional expectations and professional norms surrounding physical performance create strong social opportunity, as physical fitness is socially valued, assessed, and embedded within the study programme and future occupational role. Simultaneously, these expectations contribute to social capability by shaping shared understandings of competence and preparedness, where being physically fit is perceived as necessary for managing both operational tasks and socially demanding, high-stress situations.

### 3.9. Motivation

Motivation includes both automatic and reflective processes driving behaviour [19].

### 3.10. Automatic Motivation

Automatic motivation refers to habits and emotional responses that occur without conscious planning [19]. Several students described how modelling from their families shaped their activity habits, examplified by one participant bringing up her mom as a positive rolemodel:

*“Mom is very active, trains almost every day. […] Loves to organise hikes. I think my interest in hiking comes a lot from there.”*

Linnea

Pleasure from progress was also a central driver; experiencing strength gains and physical improvement was motivating in itself:

*“It was mainly for fun, and I wanted to get stronger. And then I noticed when I had trained and got a little more control that it became stronger […] I just thought it was really fun.”*

Freja

The students’ account show how as enjoyment and positive affect associated with feeling stronger reinforce continued participation, leading to hig levels of automatic motivation. In that way, the intrinsic pleasure derived from bodily control and strength gains appears to sustain activity through habit formation and emotional reward rather than deliberate external incentives.

### 3.11. Reflective Motivation

Reflective motivation involves conscious evaluations, goal-setting, and values [19]. Several students emphasised goals and mastery as important factors:

*“I set myself small goals that build up […] If I, for example, benchpress 80 kg this week, it’s much more motivating to say that I will do 82.5 kg next time.”*

Einar

In this manner, experiencing the process of continuous progress over time was a highly motivating factor for many of the participants.

Some students also noted that training in itself gave them a sense of mastery or achievement throughout the day, even when they faced obstacles in other areas, as one explained:

*“I feel that, even if I may not have done that much schoolwork one day, at least I’ve done something good. So, I think my motivation has actually improved.”*

Alma

Motivation was also linked to health and well-being, where exercise was used to manage stress and maintain mental balance:

*“So for mental health, it’s like, ‘OK, now I’m starting to get a little tired and lethargic. Okay, then I can just go for a jog’.”*

Bjørn

Another student explained how she uses training as a way to change scenery during busy days of studying, and for a form of mental reset:

*“If I go to school to read or work on some assignments, it’s very easy to go to the gym afterwards. Then it’s kind of a little break where you just get to relax your head completely.”*

Freja

The student’s statements reflect strong reflective motivation, as physical activity is consciously used as a strategy to manage mental fatigue, lethargy, and cognitive demands. Exercise is deliberately used as a restorative break that supports mental well-being and academic functioning, reinforcing continued engagement through perceived benefits.

Multiple students further explained how they felt that PA improved their sleep and helped them feel more refreshed the following day, further supporting reflective motivation through perceived benefits in well being:

*“You notice that when you go to bed, you sleep very well. So, you feel more rested the next day.”*

Bjørn

For some, motivation was tied to professional identity:

*“If you’re going to work as a personal trainer, I think you should both be able to train well and also actually look somewhat fit.”*

Emil

Furthermore, multiple students mentioned wanting to develop or acquire new skills as a huge factor for their motivation, as one student explained:

*“I started martial arts training one year before I moved here. I began because I wanted to... The same reason as when I started strength training. I wanted to get better at something new. And it seems quite fun. So, then I decided to continue with at least some form of martial arts after I moved here.”*

Aksel

Such description of the participants continuation training in specific activities examplify strong reflective motivation, with participation being guided by a desire for skill development, self-improvement, and meaningful challenge. The decision to sustain engagement after relocating indicates an intentional alignment between personal goals, perceived enjoyment, and ongoing commitment to physical activity.

### 3.12. Combinations of Automatic and Reflective Motivation

Several students described a mix of habitual enjoyment and conscious goal-setting, where mastery served as a strong driver:

*“When I found something I really started to master, it was fun to give myself that sense of achievement. […] It did me good, so it became very easy to prioritise it over other sports.”*

Emil

Overall, the findings show that students’ PA habits are influenced by a complex interplay of individual skills, environmental conditions, and internal drivers. Practical factors, social support, and experiences of mastery emerge as key elements for maintaining PA.

## 4. Discussion

This study examined factors that either facilitate or hinder PA among Norwegian first-year university students, interpreted through the three components of the COM-B model: capability, opportunity, and motivation [19]. The main findings indicate that students’ PA habits are shaped by a complex interplay between previous experiences, environmental conditions, and motivational processes, both automatic and reflective. Recent qualitative and mixed-methods research indicates that maintenance processes, identity, and contextual adaptation are central to understanding physical activity behaviour among university students, particularly when findings are interpreted through behaviour change frameworks such as COM-B [16]. Consistent with previous research [16,21], time constraints, financial considerations, and access to facilities emerged as key structural conditions, while social support, mastery, and progress strengthened students’ motivation to maintain regular PA.

### 4.1. The Interplay Between Physical and Psychological Capability

A central finding was the way physical and psychological capability intersect, particularly among students with prior injury experience. Several participants described how technical competence enabled them to adapt training and prevent new injuries, while simultaneously strengthening their sense of mastery. In contrast, limited knowledge or incorrect technique earlier in life was associated with injury and reduced motivation. These patterns align with research identifying skill level and self-efficacy as key drivers for sustained PA behaviour [15].

Beyond confirming the importance of both physical and psychological capability, the present findings point to specific mechanisms through which these dimensions interact. Acquiring technical knowledge and injury-management strategies appeared to reduce uncertainty and fear related to training, allowing students to reinterpret bodily signals and maintain a sense of control despite physical limitations. In this way, psychological capability may function as a regulatory mechanism that supports both self-regulation and continued engagement in physical activity.

Rather than viewing injury solely as a barrier, the ability to adapt training transformed potentially disruptive experiences into opportunities for mastery. This helps explain why several participants reported sustained or even increased motivation following injury recovery. From a COM-B perspective, enhanced capability may lower the cognitive and emotional cost of physical activity, thereby facilitating both automatic motivation through enjoyment and confidence, and reflective motivation through goal-setting and prioritisation.

Experiences of mastery when managing or recovering from an injury, for example by adapting exercises or adjusting training load, also appeared to enhance students’ cognitive capacity to regulate and plan their activities. By lowering uncertainty and perceived risk, students were able to engage more effortlessly in training, reinforcing motivation over time. These findings resonate with broader research showing that PA can be an important coping mechanism for stress. In a survey of university students in Jordan, Alkhawaldeh et al. [17] identified stress reduction as one of the main perceived benefits of PA. Similarly, Brown et al. [13] highlighted stress reduction as a core psychological outcome in their systematic review, noting that students widely believed exercise to be beneficial for mental health. Further, Radebe et al. [20] found that alleviating academic stress was a central motive for participation in physical activity among students in a semirural university context.

The present findings also align with research emphasising the role of mastery as a positive reinforcer. Pellerine et al. [19] reported that improved physical and mental health, physical appearance, and athletic performance were among the strongest motivators for PA among Canadian undergraduates; this illustrates how competence and physical improvement reinforce continued engagement. In the material from the present study, the process of regaining control over one’s body following injury reduced uncertainty and strengthened both automatic and reflective motivation, demonstrating how physical and psychological capability mutually reinforce one another.

Taken together, these findings suggest that within the COM-B model, capability cannot be understood as solely physical or psychological. Instead, it represents a dynamic resource shaped through experience, learning, and adaptation. Importantly, this dynamic contrasts with findings from studies focusing on inactive students, where injury often leads to disengagement from physical activity. Among already-active students, competence and experience may act as protective factors supporting continued participation even under challenging conditions.

### 4.2. Social Support and Training Environments as Motivational Factors

Both family and friends emerged as important sources of support for establishing and maintaining PA habits. None of the participants in the present study reported a lack of support from parents or friends as an obstacle to being physically active, in contrast to several previous studies in which limited social support has been identified as a barrier to participation [18,21]. This may reflect the fact that the sample consisted of students who were already active, a group for whom social support typically functions as a reinforcing rather than initiating factor. For these students, support appeared to strengthen existing routines and motivation rather than determine whether physical activity occurred at all.

The findings indicate that several participants did not categorise activities conducted with family—such as hiking or other outdoor-related practices—as physical activity. This label was reserved for organised or structured forms of exercise, including traditional sports, running, or strength training. This distinction is consistent with earlier research suggesting that, in the Norwegian context, *friluftsliv* or outdoor activities is commonly associated with tranquillity, simplicity, and nature experiences rather than with exercise or athletic performance, whereas physical activity and sport tend to be understood as organised, goal-oriented, and sometimes competitive practices, often taking place in constructed or deliberately modified environments [28,29]. Such culturally embedded distinctions may shape individuals’ perceptions of what “counts” as physical activity and might have implications for how activity levels are self-assessed and reported in Norwegian populations.

The findings further showed that social obligations in team sports—such as fixed training sessions and responsibilities toward teammates—were experienced as both motivating and, at times, burdensome. This suggests that social support is not uniformly beneficial but may involve a tension between commitment and autonomy. While structured social expectations increased accountability and participation, they could also contribute to pressure and reduced flexibility. This dual role of social influence aligns with the findings of Ndupu et al. [14], who describe social factors as functioning as both facilitators and barriers depending on contextual demands and role expectations.

The training environment at the university—particularly within physically demanding study programmes such as policing and sports science—was also highlighted as an important social context. Students described how peers motivated and challenged each other, contributing to a shared performance and activity-oriented culture. From a COM-B perspective, such environments can be understood as enhancing social opportunity by providing normative cues and encouragement, thereby reinforcing motivation to remain physically active. This aligns with findings by Willmott et al. [18], who demonstrating that social support is a significant indicator of opportunity within the COM-B model.

### 4.3. Structural Factors: Time, Finances, and Accessibility

Practical constraints had a substantial impact on students’ opportunities to engage in PA. Time pressure, particularly during examination periods, was highlighted as one of the most prominent barriers. This aligns with international research, where limited time is consistently identified as a major obstacle to PA among students [13,18]. Although some students managed to maintain their training routines despite academic demands, most reported that their capacity was reduced when coursework intensified. However, the present findings suggest that among already-active students, time constraints did not necessarily lead to inactivity, but instead prompted adaptations in training frequency, intensity, or type of activity. Similar patterns of adaptation and flexibility have been identified in qualitative research on physical activity habit formation among young adults, where sustained engagement was closely linked to experience, prioritisation, and integration of activity into daily routines [30]. While overall training capacity was often reduced during academically demanding periods, many students described prioritisation strategies that allowed them to maintain some level of engagement.

Geographical distance between home and training facilities was also perceived as a barrier, especially for students without access to a car. These findings are consistent with those of Brown et al. [13], who demonstrated that poor accessibility to facilities and opportunities reduces participation levels. From a COM-B perspective, such barriers can be understood as constraints on physical opportunity. Nevertheless, several students appeared to compensate for limited accessibility by choosing activities that required less travel or equipment, highlighting the role of flexibility and experience in mitigating structural constraints.

Despite having free access to training facilities through their study programmes, several students still chose to pay for additional gym memberships to increase variety in their training. This suggests that flexibility and autonomy were prioritised, even when low-cost options were available. Rather than reflecting dissatisfaction with institutional facilities, this pattern may indicate that choice and variation function as motivating factors that support sustained engagement in PA.

A practical implication of these findings is that universities should prioritise low-threshold opportunities, centralised locations for facilities, and extended opening hours. Such measures may reduce structural barriers not only by increasing access, but also by enabling students to flexibly integrate physical activity into demanding and variable study routines, as highlighted by Ndupu et al. [17]. While recommendations such as centralised training facilities and extended opening hours may reduce structural barriers Wibowo [31] and colleagues show that the effectiveness of these measures depends on the campus structure and financial framework of each university. Universities with a centralised campus may experience fewer challenges than those with distributed campuses. Accordingly, structural recommendations should be understood as context-specific examples rather than universally applicable solutions and may need to be complemented by more flexible and low-threshold strategies depending on institutional conditions.

### 4.4. Motivation as a Dynamic Process

The findings indicate that experiences of mastery may serve as a key linking mechanism between automatic and reflective motivation. As students perceive progress and competence development, enjoyment and positive affect increase, reinforcing habitual engagement. Simultaneously, this positive feedback loop supports reflective processes such as goal-setting and prioritisation of physical activity. In this way, motivation emerges not as a stable individual trait, but as a dynamic process shaped by ongoing interaction between experience, emotions, and conscious regulation. This interpretation aligns with qualitative syntheses indicating that long-term maintenance of physical activity is supported by enjoyment, identity formation, flexibility, and reflective evaluation of benefits rather than by isolated motivational triggers [32].

Students’ motivation appeared to arise from a combination of automatic processes, such as habits and emotional responses, and reflective processes, such as goal-setting and health-oriented decision-making. Many participants described PA as a source of stress relief, improved sleep, and mental balance, suggesting that psychological well-being functioned as a reinforcing outcome rather than a primary driver of behaviour. This aligns with previous research identifying psychological health benefits and goal-setting as key factors in the maintenance of PA among young adults [16,20,23,33]. Madanat and Merrill [34] showed in their cross-sectional study with college students in Jordan that motivations related to physical and mental health consistently outweighed social and recreational motives across all stages of behavioural change.

Experiences of mastery functioned as an important link between automatic and reflective motivation. When students perceived progress, they reported increases in both enjoyment—associated with automatic motivation—and their willingness to set new goals, associated with reflective motivation. This aligns with findings from Brown et al. [13], who identified goal-setting and positive reinforcement as key elements in the maintenance of PA. It also aligns with principles from self-determination theory, which emphasises that the experience of competence is fundamental for the internalisation of motivation [33].

### 4.5. Seasonal and Climatic Conditions as Barriers

Only a few participants mentioned seasonal or climatic conditions as barriers to PA; those who did described the dark winter period as reducing both motivation and activity levels. This appeared to be particularly challenging for students who had moved from southern parts of the country and were less accustomed to extended periods of limited daylight. These observations are consistent with studies showing that seasonal variation and adverse weather can influence students’ activity patterns [10,17,21]. Although this theme was not prominent across all participants, it suggests that institutions in northern regions may benefit from offering accessible options for indoor activities and increasing awareness of how seasonal fluctuations can affect motivation.

Importantly, the limited prominence of this theme suggests that seasonal conditions did not function as a decisive barrier for most participants, but rather as a contextual factor influencing motivation. From a COM-B perspective, reduced daylight may primarily affect automatic motivation through lower energy levels or mood, rather than directly constraining opportunity. Among already-active students, such seasonal challenges appeared to prompt adaptations rather than disengagement, reinforcing the role of experience and self-regulation in maintaining activity levels.

Although mentioned by relatively few participants, these findings indicate that institutions in northern regions may benefit from offering accessible indoor options and increasing awareness of how seasonal fluctuations can influence motivation, particularly for students unfamiliar with such environmental conditions.

### 4.6. Professional Identity and Body Ideals

Several students in programmes such as policing and personal training explicitly linked their engagement in PA to their future professional identity. For these students, PA appeared to be an expected component of the occupational role, potentially strengthening their motivation to maintain high levels of activity. This is consistent with findings from Ndupu et al. [14], who demonstrated that perceptions of PA as part of professional requirements vary considerably across different groups within the university sector. While some occupational groups perceive physical activity as embedded in work tasks or institutional expectations, others do not experience similar normative pressures.

This variation illustrates how professional norms and institutional expectations can shape students’ relationships with PA. In our data, this was particularly evident among students in physically demanding study programmes, where bodily functionality, performance, and preparedness were closely tied to their emerging professional identities. From a COM-B perspective, professional identity may function as a form of reflective motivation, reinforcing values, goals, and long-term commitment to physical activity.

At the same time, such expectations may contribute to the reinforcement of body ideals and perfectionistic tendencies. Previous research has shown that physical appearance is a strong motivator among young adults [22], and that appearance-related benefits often receive high priority in assessments of exercise motivation [20].Our findings suggest that similar dynamics may be present among students who view physical fitness as part of their professional legitimacy.

Taken together, these findings underscore the need for a nuanced discussion about what is understood as good physical condition within professional degree programmes. It may be particularly important for educational institutions to support the development of a sustainable and health-promoting relationship with PA, without unintentionally reinforcing unrealistic body ideals or performance-related pressures.

### 4.7. Strengths and Limitations

To the authors’ knowledge, this is the first study to investigate perceived facilitators and barriers to PA among Norwegian first-year students, using the COM-B model as an analytical framework. The study’s strong theoretical foundation provides a solid basis for future research in this area. Another strength is the rigorous analytical process, in which all interviews were independently analysed by at least two researchers; this collaborative and iterative approach helps reduce individual bias and strengthens the credibility of the findings.

Several limitations should be considered when interpreting the findings of this study. First, the sample consisted exclusively of first-year students. This group is situated in a transitional phase characterised by adaptation to new academic demands, social environments, and increased responsibility for self-care and daily structure. Compared to students in later years of higher education, first-year students may therefore encounter distinct challenges and opportunities related to physical activity. Accordingly, the findings primarily reflect factors relevant to the transition into higher education and should not be uncritically generalised to students in later stages of their studies. Second, the sample consisted of a relatively small and context-specific group of participants. The study included 15 first-year students recruited from a university campus located in a more rural region of Northern Norway. As such, the findings cannot be assumed to be statistically generalisable to the broader population of university students, particularly those studying in larger urban institutions or in other national contexts or climate. However, recruitment was continued until data saturation was reached, suggesting that the material was sufficiently rich to capture central facilitators and barriers to physical activity within this specific group. The analytical themes may therefore be considered transferable to other physically active students studying at rural or regionally similar universities in northern contexts, where institutional structures, climate, and campus environments may resemble those described in this study.

A third limitation relates to potential selection bias in the recruitment process. Although the sampling strategy initially aimed to include both physically active and inactive first-year students, only students who were already physically active ultimately chose to participate in the qualitative interviews. This self-selection may have shaped the findings, as participants likely had more positive attitudes toward physical activity and greater experience navigating barriers to engagement. Consequently, the results primarily reflect factors that support the maintenance of physical activity rather than those associated with initiating activity among inactive students. At the same time, this limitation also represents an analytical strength, as it allowed for a more focused exploration of sustaining mechanisms for physical activity—an area that has received comparatively less attention in the existing literature. Nonetheless, caution should be exercised when applying these findings to less active or inactive student populations, whose perceived barriers and motivational processes may differ substantially.

Finally, it should also be considered that the interview guide did not include explicit or systematic questions specifically targeting barriers and facilitators to physical activity across all domains, which may have resulted in variation in the depth and consistency with which certain influences were explored across interviews.

## 5. Conclusions

In conclusion, the present study shows how intertwined certain components of the COM-B framework are in relation to facilitating or hindering participation in PA among Norwegian first-year students. Aspects such as technical competence, injury management, effective self-regulation, access to facilities, and support from friends and peers were highlighted. Future research should investigate how these results can inform institutional structures to better support sustainable activity habits among college and university students.

## Figures and Tables

**Table 1:** Main topics and questions from the interview guide.

Topic	Questions
Background	How old are you? Where did you grow up? What study program are you enrolled in?
Sports/Physical activity biography	What kind of sports/activities have you been involved in growing up?
Sports/Physical activity throughout the first academic year	What kind of sports/physical activity are you involved in now? How did your activity patterns evolve/change throughout the academic year? What are your goals regarding the activities you participate in? Does the study you are enrolled in or your future occupation effect your activity patterns and/or motivation? How do you think your participation patterns will evolve in the future?

**Table 2:** Overview of the interview analysis process with selected examples.

Original Quote	Condensation	Theme	Category Within the COM-B Framework [19]
*So, I think it’s quite easy to train parallel to my studies, because there aren’t that many study hours we have in a week. It’s not like having worked after four, so it’s my free time. So, I think it’s easy to get a workout into the day.*	The student finds it easy to fit in training because their studies leave sufficient free time, due to few obligatory teaching hours throughout the week.	Study-related obligations	Physical opportunity
*An active childhood. With football and football. The whole family played football at the same club. So there has been a community with that football.*	An active childhood shaped by family involvement in football.	Family preferences	Social opportunity
*I want to be able to look at myself like that, I feel stronger. I think it’s very fun to kind of increase muscles, no, increase weight.*	Feeling stronger and increasing weights in training is motivating and enjoyable	Perception of results and progression	Automatic motivation

**Table 3:** Overview over the subthemes within the different domains of the COM-B model present in the data material (themes in alphabetical order within every domain).

Topi	Questions
**Capability**	
Physical capability	Injuries or illness as an interruption/disruption of routines. Overload, overuse injuries or chronic injuries/conditions stop activity.
Psychological capability	Perceptions of what constitutes a high level of activity. Insufficient or lack of training supervision/knowledge leading to injury. Competence built through school and further education. Competence to take responsibility/prioritize training. Adjusting to the new life as a student.
Combination of physical and psychological capability	Experience with injuries from the past leading to competence in handling new/reoccurring injuries.
**Opportunity**	
Physical opportunity	Access to facilities. Activities offered on campus. Physical distance to activity/facilities. Financial constraints. Weather and climate. Prioritisation due to tight time schedule / different obligations.
Social opportunity	Experience of a sense of community in training. Parents as facilitators during upbringing. Activity level and common activities in the family during upbringing. Pushing each other in training. Impact of the activity level of the environment/peer group.
Combination of physical and social opportunity	Specific requirements within the study, job or future occupation. Coordination of study/school work and other activities.
**Motivation**	
Automatic motivation	Active family members as a role model. Fun/enjoyment in achieving results/progress.
Reflective motivation	Working towards a specific goal. Belief that PA affects overall health and well-being both physically and mentally. PA to promote better sleep. Maintaining role image. Desire to learn new skills.
Combination of reflective and automatic motivation	Sense of mastery.

## Data Availability

The datasets presented in this article are not readily available, due to privacy restrictions.
